# Biological Safety Studies and Simultaneous Determination of Linagliptin and Synthetic Impurities by LC-PDA

**DOI:** 10.1155/2019/7534609

**Published:** 2019-03-03

**Authors:** Raquel Balestri Heleno Ferreira, Jonathaline Apollo Duarte, Flávio Dias Ferreira, Luis Flávio Souza de Oliveira, Michel Mansur Machado, Marcelo Donadel Malesuik, Fávero Reisdorfer Paula, Martin Steppe, Elfrides Eva Shermann Schapoval, Clésio Soldateli Paim

**Affiliations:** ^1^Laboratório de Pesquisa em Desenvolvimento e Controle de Qualidade, Universidade Federal do Pampa, Uruguaiana, RS, Brazil; ^2^Programa de Pós-Graduação em Ciências Farmacêuticas, Universidade Federal do Pampa, Uruguaiana, RS, Brazil; ^3^Núcleo de Pesquisa em Bioquímica, Toxicologia e Imunologia, Universidade Federal do Pampa, Uruguaiana, RS, Brazil; ^4^Programa de Pós-Graduação em Ciências Farmacêuticas, Universidade Federal do Rio Grande do Sul, Porto Alegre, RS, Brazil

## Abstract

A stability-indicating LC method was developed for quantification of linagliptin (LGT) and three synthetic impurities. The method utilizes a Thermo Scientific® RP-8 column (100 mm × 4.6 mm; 5 *μ*m) with the PDA detector for quantitation of impurities. A mixture of 0.1% formic acid with pH 3.5 (A) and acetonitrile (B) was used as the mobile phase at a flow rate of 0.6 mL·min^−1^ with gradient elution. The percentage of mobile phase B increases from 30% to 70% over 5 min and decreases from 70% to 30% between 5 and 8 min. The method was validated according to International Council for Harmonization (ICH) guidelines. The LOD values obtained were 0.0171 *μ*g·mL^−1^ and 0.015 *μ*g·mL^−1^ for LGT and impurities, respectively. The LOQ values were 0.06 *μ*g·mL^−1^ for LGT and impurities. In all cases, the correlation coefficients of LGT and impurities were >0.999, showing the linearity of the method. The % recovery of the LGT and added impurity were in the range of 92.92–99.79%. The precision of the method showed values less than 1.47% for LGT and less than 4.63% for impurities. The robustness was also demonstrated by small modifications in the chromatographic conditions. The selectivity was evidenced because the degradation products formed in stress conditions did not interfere in the determination of LGT and impurities. Toxicity prediction studies suggested toxicity potential of the impurities, which was confirmed using biological safety studies in vitro.

## 1. Introduction

Diabetes mellitus is a chronic, nontransmissible metabolic disease that affects approximately 422 million individuals worldwide, with a projected doubling over the next 20 years according to the World Health Organization [1]. Among types, the type 2 mellitus diabetes is more common and is more likely to increase in the population. This disease is characterized by insulin secretion disorder, insulin resistance in target tissues, or receptor desensitization of insulin [2].

Linagliptin (LGT) is an orally active and competitive DPP-4 enzyme inhibitor used to treat type 2 diabetics which failed to achieve glycemic control with metformin alone [3]. The drug is administered as a single drug once daily as a 5 mg dose in adults and/or in combination with metformin or empagliflozin [4, 5]. The drug is approved in the US, Europe, Japan, Brazil, and other countries [6].

The literature reviewed presents a series of studies for quantification of the drug LGT in biological fluids [7–9] and in pharmaceutical forms [10–14] and characterization of synthetic impurities [15, 16]; however, no studies have been described to quantify the drug in the presence of its main synthetic impurities. Moreover, biological safety studies of the drug containing the impurities and in silico studies of toxicity are also not described.

**Figure 1:**
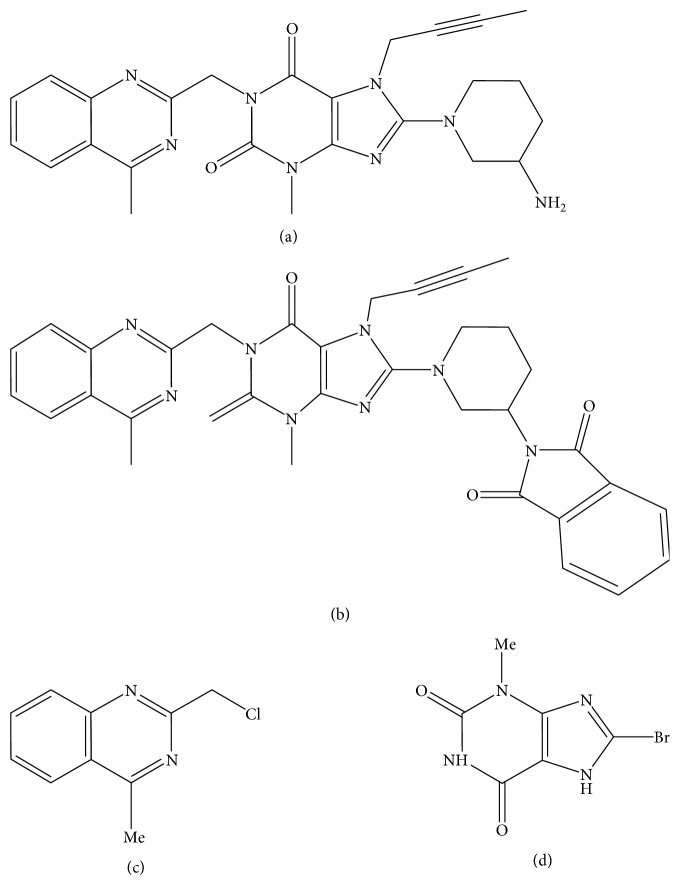
Chemical structures of LGT (a) and its synthetic impurities (b)–(d).

However, with the premise that the drugs used in the pharmaceutical formulation process are not considered totally pure and that the presence of these impurities, even in small quantities, can influence the efficacy and safety of pharmaceuticals, it is opportune to develop and validate a LC method to quantify the drug LGT (Figure 1(a)) in presence of its impurities of synthesis (Figures 1(b)–1(d)) and to evaluate the biological safety of these substances using cytotoxicity and genotoxicity assays. Besides that, the toxicity of LGT and its impurities was evaluated against different software in order to predict whether these molecules present some risk of causing a mutagenic effect to biological cells. These results may assist in understanding the mechanisms of possible toxic effects caused by these molecules.

## 2. Experimental

### 2.1. Chemicals

The reference chemical substances of the LGT drug ({1H-purine-2,6-dione, 8-[(3R)-3-amino-1-piperidinyl]-7-(2-butyn-7-dihydro-3-methyl)-1-[(4-methyl-2-quinazolinyl)methyl]) (CAS: 668270-12-0; 99.9%) and the impurities of synthesis: impurity 1 (8-bromo-3,9-dihydro-3-methyl-1H-purine-2,6-dione) (CAS: 93703-24-3; 99.9%), impurity 2 (2-(chloromethyl)-4-methylquinazoline) (CAS: 109113-72-6; 99.9%), and impurity 3 (7-(2-butynyl)-8-[(3R)-3-(1,3-dihydro-1,3-dioxo-2H-isoindol-2-yl)-1-piperidinyl]-3,7-3-methyl-1-[(4-methyl-2-quinazolinyl)methyl]-1H-purine-2,6-dione); R-(7-(But-2-yn-1-yl)-8-(1,3-dioxoisoindolin-2-yl)piperidin-1-yl)-3-methyl-1-((4-methylquinazolin-2-yl)methyl)-1H-purine-2,6(3H,-dione) (CAS: 886588-63-2; 99.9%) were purchased from Sequoia Products (Pangbourne, UK). Commercial samples of the drug Trayenta®, produced by Boehringer Ingelheim and containing 5 mg of LGT in coated tablets, were obtained in a local market (Uruguaiana, RS, Brazil). The excipients contained in the dosage form (mannitol, pregelatinized starch, starch, magnesium stearate, copovidone, and Opadry® pink) were all pharmaceutical grades and were acquired from different suppliers. Acetonitrile and methanol of HPLC grade were purchased from Tedia® (Fairfield, USA) and phosphoric acid from Merck® (Darmstadt, Germany). Hydrochloric acid (Proquímios®, Rio de Janeiro, Brazil), formic acid (Vetec®, Duque de Caxias, Brazil), ammonium hydroxide (Proquímios®, Rio de Janeiro, Brazil), sodium hydroxide (Dinâmica®, Diadema, Brazil), hydrogen peroxide Merck® (Merck®, Darmstadt, Germany), and dimethylsulfoxide (Vetec®, Duque de Caxias, Brazil) were of analytical grade. Purified water was obtained from Elga Equipment (High Wycombe, UK).

### 2.2. Apparatus

A Thermo Scientific Dionex Ultimate® 3000 liquid chromatograph (Thermo Scientific Dionex Ultimate® 3000, Waltham, MA, USA) equipped with a model LPG-3400SD quaternary pump, WPS-3000TSL autosampler, TCC 3000RS column oven, photodiode-array detector (DAD-3000), and Chromeleon 6.8 (Waltham, MA, USA) manager system software was used. Photodegradation studies were carried out in a photostability UV chamber (1.0 × 0.17 × 0.17 m) with mirrors and equipped with UV-A (Light Express®, 352 nm, 30 W).

### 2.3. Chromatographic Conditions

The chromatographic separation was performed in a Thermo Scientific® RP-8 column (150 × 4.6 mm ID, 5 *µ*m, Waltham, MA, USA). The mobile phase comprises a mixture of (A) 0.1% (v*/*v) formic acid (pH adjusted to 3.5 with ammonium hydroxide) and (B) acetonitrile at a flow rate of 0.6 mL·min^−1^ with gradient elution. The percentage of mobile phase B increases from 30% to 70% over 5.0 min and decreases from 70% to 30% between 5.0 and 8.0 min. The injection volume was 20 *µ*L for both reference substance and drug product solutions. The temperature was set at 30°C in the column oven. LGT was determined by UV detection at 294 nm using photodiode array (DAD) and IMP 1, IMP 2, and IMP 3 at 278 nm, 268 nm, and 280 nm, respectively.

### 2.4. Standard and Sample Preparations for LC Analysis

Sample stock solution of LGT (200.0 *µ*g·mL^−1^) was freshly prepared in a 50 mL volumetric flask by dissolving the equivalent of 10.0 mg of LGT from tablets in methanol. Stock standard solution of LGT (200.0 *µ*g·mL^−1^) was freshly prepared in a 25 mL volumetric flask by dissolving 5.0 mg of standard LGT in methanol. Impurities standard stock solution of LGT (200.0 *µ*g·mL^−1^) was freshly prepared in a 25 mL volumetric flask by dissolving 5.0 mg of standard LGT in methanol. All solutions were prepared individually and stored in an amber bottle under refrigeration at 8°C.

### 2.5. Validation of the Analytical Method by LC

The validation of the analytical method for quantification of the LGT drug and its main synthetic impurities was performed according to the International Conference on Harmonization [17–19] by analyzing the following analytical parameters: specificity and selectivity, detection and quantification limits, linearity, precision, accuracy, and robustness.

#### 2.5.1. Specificity

Forced degradation studies were performed for LGT sample solution (200 *μ*g·mL^−1^) to provide an indication of the stability-indicating property and specificity of the proposed method. The excipient interference was also evaluated, and all solutions used in the assays were protected from light. Blank solutions were used during the analysis. For the peak purity test, a photodiode array detector (PDA) was used and the purity factor was also evaluated as a support to analyze peak homogeneity. The forced degradation studies were performed in triplicate in the following conditions:

*(1) Acid Hydrolysis*. One milliliter of the LGT stock solution (200.0 *μ*g·mL^−1^) was transferred to a 10 mL volumetric flask, 2.0 mL of 1.0 mol·L^−1^ HCl was added, and the solutions were stored at room temperature (23 ± 1°C) for 24 and 48 h. Subsequently, the solutions were neutralized with 1.0 mol·L^−1^ NaOH, and 180 *μ*L of the impurities stock solution (200.0 *μ*g·mL^−1^) was added and diluted in the mobile phase to achieve a concentration of 20.0 *μ*g·mL^−1^ of LGT and 3.6 *μ*g·mL^−1^ of each impurity.

*(2) Acid Hydrolysis at 80°C*. The same procedure described in “*Acid Hydrolysis*” was performed at 80°C for 2, 4, 6, and 10 hours to verify the degradation of the drug in these conditions.

*(3) Basic Hydrolysis*. One milliliter of the LGT stock solution (200.0 *μ*g·mL^−1^) was transferred to a 10 mL volumetric flask, 2.0 mL of 1.0 mol·L^−1^ NaOH was added, and the solutions were stored at room temperature (23 ± 1°C) for 24 and 48 h. Subsequently, the solutions were neutralized with 1.0 mol·L^−1^ HCl, and 180 *μ*L of the impurities stock solution (200.0 *μ*g·mL^−1^) was added and diluted in the mobile phase to achieve a concentration of 20.0 *μ*g·mL^−1^ of LGT and 3.6 *μ*g·mL^−1^ of each impurity.

*(4) Basic Hydrolysis at 80°C*. The same procedure described in “*Basic Hydrolysis*” was performed at 80°C for 15, 30, 45, 60, 90, and 120 minutes to verify the degradation of the drug in these conditions.

*(5) Oxidative Degradation*. One milliliter of the LGT stock solution (200.0 *μ*g·mL^−1^) was transferred to a 10 mL volumetric flask, 2.0 mL of 30% (v/v) hydrogen peroxide solution was added, and the solutions were store at room temperature (23 ± 1°C) for 2, 4, 6, and 10 h. Subsequently, 180 *μ*L of the impurities stock solution (200.0 *μ*g·mL^−1^) was added and diluted in the mobile phase to achieve a concentration of 20.0 *μ*g·mL^−1^ of LGT and 3.6 *μ*g·mL^−1^ of each impurity.

*(6) Thermal Degradation*. Five milliliters of the LGT stock solution (200.0 *μ*g·mL^−1^) was exposed at 60°C in an oven for 1, 2, and 3 h. Subsequently, the total volumes of the flask were transferred to 50 mL volumetric flask, and 180 *μ*L of the impurities stock solution (200.0 *μ*g·mL^−1^) was added and diluted in the mobile phase to achieve a concentration of 20.0 *μ*g·mL^−1^ of LGT and 3.6 *μ*g·mL^−1^ of each impurity.

*(7) Photodegradation*. One milliliter of the LGT stock solution (200.0 *μ*g·mL^−1^) was exposed to UV-A radiation (352 nm) for 1, 2, 3, and 6 h. The stress degradation study was performed by exposing the solutions in quartz cells in the photodegradation chamber, where the samples were positioned horizontally to provide the maximum area of exposure to the light source. Subsequently, the total volumes of the flask were transferred to the 10 mL volumetric flask, and 180 *μ*L of the impurities stock solution (200.0 *μ*g·mL^−1^) was added and diluted in the mobile phase to achieve a concentration of 20.0 *μ*g·mL^−1^ of LGT and 3.6 *μ*g·mL^−1^ of each impurity. Control samples were protected from light with the aluminum foil and were also placed in the light chamber and exposed concurrently.

#### 2.5.2. Detection Limit (LOD) and Quantitation Limit (LOQ)

LOD and LOQ of the drug using the LC method were obtained based on the signal-to-noise approach. The background noise was obtained after injection of the blank solution containing a mixture of methanol and 0.1% formic acid with pH 3.5 (50 : 50 v/v), observed over a distance equal to 20 times the width at half-height of the LGT (120.0 *μ*g·mL^−1^) and impurities peaks (0.180 *μ*g·mL^−1^). LOD and LOQ values were experimentally determined with six replicates using the signal-to-noise ratio of 3 : 1 and 10 : 1, respectively.

#### 2.5.3. Linearity

Three calibration curves were prepared with six concentrations in the ranges of 2.42 to 144.0 *µ*g·mL^−1^ of LGT RS and 0.060 to 3.6 *µ*g·mL^−1^ of each synthetic impurity. For each concentration, solutions were prepared and injected in triplicate. The peak areas of the chromatograms were plotted against the respective concentrations of the drug and synthetic impurities to obtain the analytical curves. The calculation of the regression line was employed by using the method of least squares, and the curves were validated through analysis of variance. The concentration ranges were determined using the limits established by the ICH Q3A (R2) regulatory guide [19].

#### 2.5.4. Precision

Precision was determined using the parameters of repeatability (intraday) and intermediate precision (interday), analyzing six LGT sample solutions prepared at 120.0 *µ*g·mL^−1^ containing the synthetic impurities at 0.180 *µ*g·mL^−1^. The assays were performed in triplicate in three different days, and the results were expressed as relative standard deviation (RSD) of the analytical measurements.

#### 2.5.5. Accuracy

The accuracy was determined by the recovery of known amounts of LGT reference standard and impurities (1, 2, and 3) added to the placebo solution. The added levels were 75, 100, and 125% of the nominal drug concentration (120 *µ*g·mL^−1^) and 0.125, 0.25, and 0.5% of the impurities in relation to the nominal concentration of the drug. The results were expressed as the percentage of LGT reference standard and impurities recovered.

#### 2.5.6. Robustness

The robustness of the analytical method was performed in order to evaluate the susceptibility of measurements due to deliberate variations in analytical conditions. It was determined by analyzing the standard and sample solutions with the following deliberate changes to the chromatographic conditions: column temperature (±5°C), flow rate (±0.03 mL·min^−1^), mobile phase with pH (±0.2 unit) variation and wavelength of LGT, and synthetic impurities (±3 nm). The evaluation of the results was carried out from the application of Student's *t*-test, comparing the average percentage of analytes in each parameter with the average percentage of analytes in the nominal chromatographic conditions.

### 2.6. Biological Safety Studies

The white blood cell cultures were prepared using a blood sample collected from a volunteer donor, according to procedures approved by the Research Ethics Committee of the Federal University of Santa Maria (letter of approval no. 27045614.0.0000.5323). Venous blood was immediately diluted in RPMI 1640 supplemented with 10% fetal bovine serum, 5% phytohemagglutinin, sodium bicarbonate 2 g·L^−1^, and 1% streptomycin/penicillin at the proportion of 1 : 1 (v/v). The cells were placed in an incubator at 37°C and 5% CO_2_ for 72 hours [20]. For the tests, four groups were used, and the negative control received PBS 7.4 and the positive control 100 mmol·L^−1^ hydrogen peroxide.

The concentrations used in the experiments were obtained from pharmacokinetic data of the drug concentration in the plasmatic peak (0.393 ng·mL^−1^) described by Neumiller and Setter [21]. The concentrations ranged between 3.93 ng·mL^−1^ (10 times the peak plasmatic concentration) and 0.0393 ng·mL^−1^ (0.1 times the peak plasmatic concentration) of the drug in PBS 7.4 buffer. In all conditions, the equivalent to 1.0% of each impurity was added to the LGT solutions. All assays were performed in triplicate.

The study of cellular viability was evaluated by the loss of membrane integrity using the trypan blue test. Cells exposed to the dye were analyzed microscopically in a Neubauer chamber, and the results were expressed as percentage of the control value [22].

Cell proliferation analyses were also performed in a Neubauer chamber to differentiate between living and dead cells, observing the blue colour of the dead cells using Turk's solution as a dye (acetic acid 3% and 1% gentian violet in water) [23].

The comet assay was performed according to Montagner and collaborators [20] and Tice and collaborators [23]. One hundred cells per slide were counted in triplicate for each group. Cells were classified according to the length of their tail, and the levels of damage were from 0 (no damage) to 4 (maximum damage).

The micronucleus test was carried out according to the technique described by Schmid [24], which allows the identification of increased frequency of micronuclei in cells exposed to genotoxic agents, which express damage in their chromosomes. All analyses were performed using toxicity specific statistical software. Data were evaluated by ANOVA followed by post hoc Bonferroni. Results were considered significant at *p* < 0.05.

### 2.7. Computational Toxicology

The chemical structure of LGT and the impurities described by the SMILES language were used to evaluate the toxicity prediction for the AMES test and potential tumorigenic effect with the use of pKCSM, Osiris Property Explorer, and LAZAR computer programs available online.

## 3. Results and Discussion

The LC procedure was optimized to develop a stability-indicating method to separate the LGT drug, degradation products obtained in stress conditions, and three synthetic impurities with good system suitability value. The chromatographic conditions were chosen after the test of different stationary phases (C8 and C18) and mobile phases with distinct ratios of organic solvent (acetonitrile or methanol) and water, with and without buffer solutions at different pH values. Initial studies were performed with the isocratic elution mode; however, the separation was not efficient with 50% acetonitrile (*R* < 1.0), or the analysis time was very high (40 minutes) with 30% acetonitrile. The use of acetonitrile as an organic component resulted in a sufficient resolution (*R* > 2) between LGT, degradations products and synthetic impurities, and symmetric peaks in comparison with methanol using the gradient mode. Different pH values were used during the development of the analytical method. The best results of symmetry and resolution were obtained using 0.1% (v*/*v) formic acid (pH adjust to 3.5). Other pH values were also evaluated (4.0 and 5.0), but these pH values promote an increase in the peak asymmetry (As > 3.0). The use of buffered solutions did not promote improvements in analytical conditions that were not necessary, facilitating the cleaning process of the column and equipment. Finally, a mobile phase comprising a mixture of (A) 0.1% (v*/*v) formic acid (pH adjusted to 3.5 with ammonium hydroxide) and (B) acetonitrile at a flow rate of 0.6 mL·min^−1^ with gradient elution was adopted. The percentage of mobile phase B increases from 30% to 70% over 5 min and decreases from 70% to 30% between 5 and 8 min.

**Figure 2:**
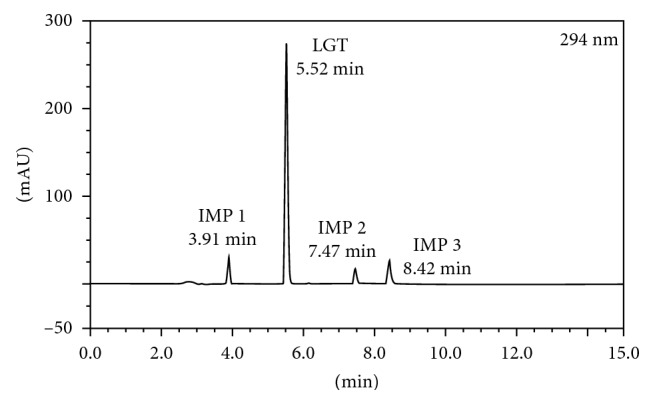
Chromatogram obtained for linagliptin and impurities under optimized chromatographic conditions: IMP 1 (impurity 1 at 3.91 min); LGT (linagliptin at 5.52 min); IMP 2 (impurity 2 at 7.47 min); IMP 3 (impurity 3 at 8.42 min).

To improve the sensitivity of the method, each substance was determined in its wavelength of maximum absorption. LGT was determined by UV detection at 294 nm using photodiode array and the synthetic impurities 1, 2, and 3 at 278 nm, 268 nm, and 280 nm, respectively. Retention times were 3.91, 5.52, 7.47, and 8.42 min for impurity 1, LGT, impurity 2, and impurity 3, respectively (Figure 2), allowing rapid determination of the substances.

The parameters of system suitability obtained in the conditions developed for the analytical method are described in Table 1. According to these results, the LC system and procedure showed that they are capable to provide data of acceptance quality [25, 26].

### 3.1. Method Validation

#### 3.1.1. Selectivity

**Figure 3:**
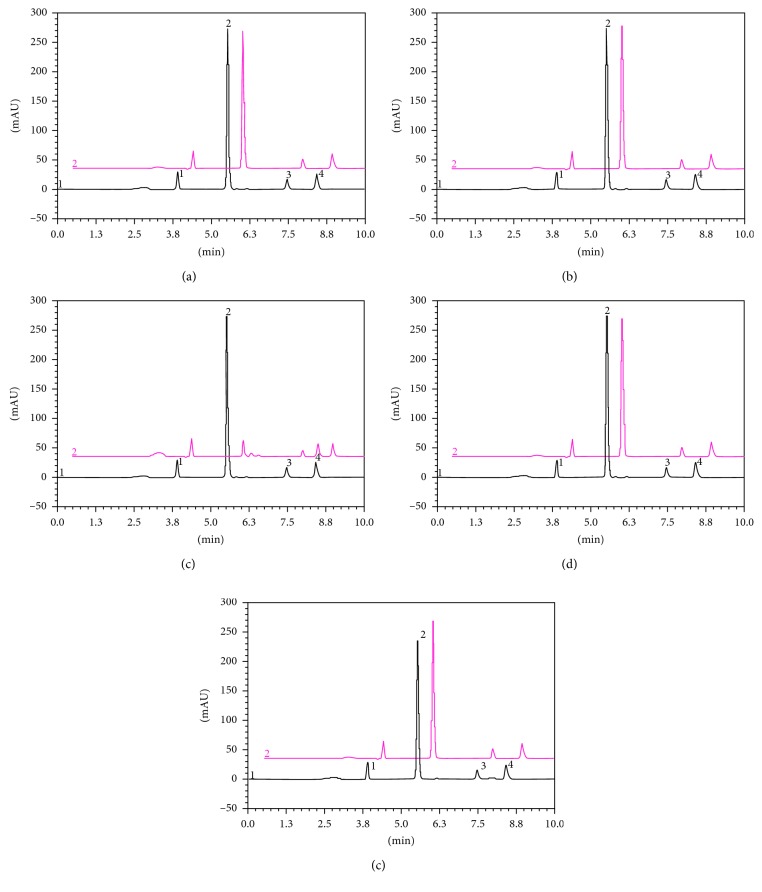
Chromatograms obtained during specificity study. (a)–(e) Drug product (20 *µ*g·mL^−1^): (a) photodegradation (UVA, 4 h); (b) oxidative degradation (30% H_2_O_2_); (c) basic hydrolysis (1.0 mol/L NaOH) at 80°C; (d) acid hydrolysis (1.0 mol/L HCl) at 80°C; (e) dry heat at 60°C. Impurity 1 (peak 1; 3.91 min); LGT (peak 2; 5.52 min); impurity 2 (peak 3; 7.47 min); impurity 3 (peak 4; 8.42 min).

The evaluation of the selectivity of the analytical method demonstrated greater instability of the LGT drug under alkaline conditions (1.0 mol·L^−1^ NaOH and 1.0 mol·L^−1^ NaOH at 80°C) with a reduction of 23.19 and 86.57% at drug concentration, respectively (Table 2). In this condition was verified one additional peak detected at 8.0 min (Figure 3).

Under the acid (1 mol·L^−1^) condition, no significant decrease in area of LGT was observed. However, when the drug was exposed to acid condition and temperature, the drug has a degradation of the 15.50 at 10 h. In photolytic conditions (UVA), the LGT in methanolic solution degraded 34.56% in 4 h, showing the instability of drug. However, there was no corresponding formation of degradation products in the conditions of analysis. In accordance with Bakshi and Singh [27], such a situation could be due to drug decomposition into low-molecular weight fractions or due to the formation of nonchromophoric degradation products. Furthermore, the drug also proved unstable under other stress conditions, as shown in Table 2.

In all stress conditions evaluated, the purity of LGT and impurities peaks was verified, demonstrating that there were no other substances coeluting at the same retention time.

In addition, the selectivity of the method was also determined by checking the interference of the formulation excipients in the detection and quantification of LGT and its synthetic impurities. The results showed that there was no interference of the placebo with the chromatographic peaks of interest.

The chromatographic peak purity tool was applied to all LGT and synthetic impurities peaks and demonstrated that they were pure in all cases, confirming the absence of other substance coeluting in the same retention times. Since the main peak of LGT was not found attributable to any other substance, the method proves to be a stability indication.

#### 3.1.2. Linearity

To assess linearity, the curves for LGT and impurities were constructed by plotting concentration (*μ*g·mL^−1^) versus area (mAU). The correlation coefficients were 0.9996, 0.9963, 0.9994, and 0.9991 for LGT and impurities 1, 2, and 3, respectively, which indicated excellent linearity. The analysis of variance was applied to verify the linearity of the method, and the results showed that the regression equations were linear (*F* calculated > *F* critical; *λ* = 0.05) with no deviation from linearity (*F* calculated < *F* critical; *λ* = 0.05) in all cases. Student's *t*-test was performed to verify the significance of the experimental intercepts in the regression equation. According to the results, it is not significantly different from the theoretical zero value for *p* > 0.05 in all cases.

#### 3.1.3. Detection Limit (LOD) and Quantitation Limit (LOQ)

For sensitivity experiments, the limit of detection (LOD) and limit of quantitation (LOQ) were estimated at a signal-to-noise ratio of approximately 3 : 1 and 10 : 1, respectively [28]. The results indicated that the LOD were 0.0171 *μ*g·mL^−1^ for LGT and 0.0156 *μ*g·mL^−1^, 0.0147 *μ*g·mL^−1^, and 0.0168 *μ*g·mL^−1^ for the impurities 1, 2, and 3, respectively. The result of LOQ for LGT was 0.057 *μ*g·mL^−1^ and for impurities 1, 2, and 3 were about 0.06 *μ*g·mL^−1^ in all cases.

#### 3.1.4. Precision

Intraday and interday precision results are expressed as relative standard deviations (RSD%). The results are presented in Table 3 for both the repeatability and the intermediate precision. The variability of the results was low with RSD% values less than 2% to intraday and 1.36% to interday for quantitative determination of LGT drug and less than 20% to intraday and interday for quantitative determination of the synthetic impurities, indicating the precision of the developed method [28].

#### 3.1.5. Accuracy

The data for accuracy were expressed in terms of LGT and synthetic impurities recoveries percentage from the known quantities added to the placebo solution. For each level of LGT and synthetic impurities concentration, three determinations were performed. The mean recovery data were within the range of 99.65 to 99.98% to LGT drug and range of 87.34 to 99.34%. These results satisfying the acceptance criteria for the study, where the recovery percentage to drugs should be in the range of 98 to 102% and to synthetic impurities, should be in the range of 80 to 120%. The values of RSD% were also within the specified values (less than 1% for drugs and less than 20% for impurities), according to the literature [28].

#### 3.1.6. Robustness

The robustness evaluation was performed by quantifying the LGT drug and its synthetic impurities using small modifications in the chromatographic conditions of the analytical method. The results obtained (Table 4) demonstrated that the modifications performed did not significantly alter the quantification of LGT drug and synthetic impurities (*p* > 0.05).

### 3.2. Biological Safety Studies

**Figure 4:**
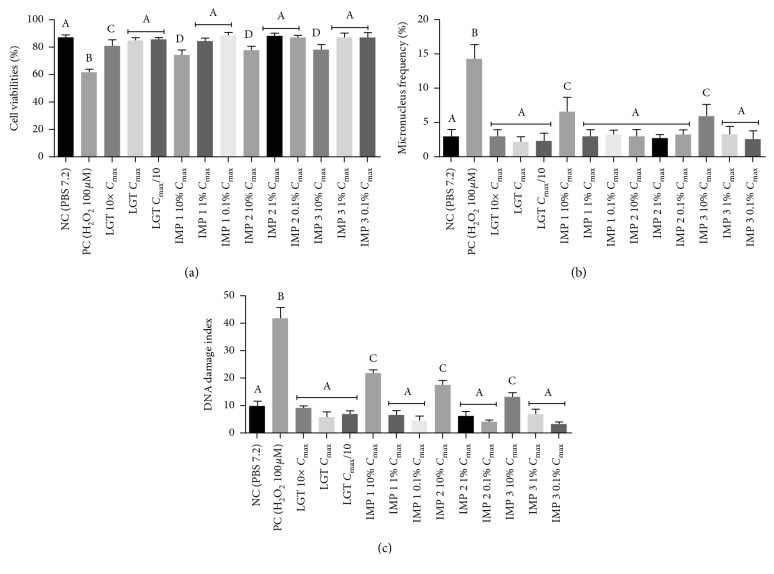
Results of the biological safety studies of the LGT drug and synthetic impurities in different concentrations. (a) Cellular viability; (b) micronucleus frequency; (c) DNA damage. NC: negative control; PC: positive control; LGT: linagliptin; IMP 1: impurity 1; IMP 2: impurity 2; IMP 3: impurity 3; 10 × *C*_max_: ten times greater than the maximum concentration of LGT; *C*_max_: maximum concentration of LGT; *C*_max_/PP: ten times lower than the maximum concentration of LGT. ^*∗*^Different letters differ statistically.

The cytotoxic, genotoxic, and mutagenic parameters performed in the biological safety analysis of LGT drug and synthetic impurities were evaluated through cell viability, comet test, and micronucleus frequency tests. In Figure 4(a), it can be seen that LGT reduced cell viability at a concentration 10 times greater than the plasma concentration (3.93 ng·mL^−1^) and the impurities reduced at a concentration equivalent to 10% of the maximum plasma concentration of the drug (0.0393 ng·mL^−1^).

The results showed that all impurities in the concentration equivalent to 10% of the maximum plasma concentration of the LGT showed mutagenic and genotoxic activity, evidencing a possible relation with malformations, congenital diseases, genetic and degenerative diseases, cellular aging, and malignant neoplasm, among others [29], showing the importance of quality control and the need for qualitative and quantitative determination of the synthetic impurities in the pharmaceutical products containing the LGT drug.

### 3.3. Toxicity Prediction Studies

The computational programs pKCSM and Osiris Property Explorer use determination of physicochemical properties and analysis of structural similarity or molecular fragments with therapeutic or nontherapeutic compounds with recognized toxicity from their databases to provide results for toxicity prediction.

LAZAR software is automated and reproducible in silico toxicology software used as a comparison to experimental results. This program is used to predict the potency (TD50) of potential carcinogenic agents from fragments of molecules under study and their structural similarity to known or genotoxic or carcinogenic chemical groups or compounds. This analysis takes place from the use of experimental data found in databases, such as the distributed structure-searchable toxicity (DSSTox) databases (http://www.epa.gov/ncct/dsstox/) and the comparison with the study molecules. The data of the similarity are submitted to mathematical (Q)SAR models of the genotoxic and carcinogenic effects aiming at performing the prediction of their potential toxicity. For efficiency reasons, only instances of the set molecules (training set) with a similar preset threshold above 0.3 were considered to be analogous to the structure of the compounds [30–34]. All predicted results of the potential risk of toxicity are shown in Table 5.

From the results obtained, it was verified that IMP 2 presented prediction of mutagenicity with the use of all software used, moreover two suggestions of the carcinogenic effect with the use of Osiris and LAZAR. Likewise, the pKCSM program has suggested that LGT and IMP 1 and IMP 2 have shown indication of mutagenicity, which the potentially toxic effect may be by the amino, bromine, and chlorine moieties attached in the chemical structures, respectively. The LAZAR software using (Q)SAR models suggested a potential indication to the toxic effect of LGT and IMP 1 and IMP 3 could not be predicted by the applicability of the domain model, which together with the results of the other software's suggest the absence of potential toxicity. Computer programs do not assess the toxic dose-response relationship and qualitatively describe the potential risk of toxicity of these molecules according to their different databases. From these results, it is suggested the molecules with a positive result can have a toxic effect according to their chemical structures.

In considering the experimental results obtained from Comet assay, the IMP 1 and IMP 2 present DNA damage at concentrations of 10 × *C*_max_, which is in accordance with the suggestion of the in silico studies. Only IMP 3 did not present a similar result to the theoretical study, which may be indicative of absence of a functional group characterized as toxic by all programs.

## 4. Conclusion

A stability-indicating LC method was validated to quantify LGT and its main impurities in coated tablets. The method proved to be simple, linear, accurate, precise, and robust and can be applicable to quantitative determination of LGT and synthetic impurities in routine analysis. Toxicity prediction studies suggested toxicity potential of the impurities, which was confirmed using biological safety studies in vitro. The results confirmed the need to quantify the synthetic impurities in the commercial formulations.

## Figures and Tables

**Table 1:** Parameters of system suitability of the analytical method developed and results recommended by the FDA [25].

Parameter	Recommended FDA [22]	IMP 1	LGT	IMP 2	IMP 3
Retention time (min)	—	3.91	5.52	7.47	8.42
Retention factor (*k*)	*k*′ > 2	2.03	3.27	4.79	5.52
Tailing factor (*T*)	*T* ≤ 2	1.06	1.20	1.14	1.19
Efficiency (*N*)	*N* > 2000	5666	8466	13391	13234
Resolution (*R*)	*R* > 2	—	3.63	3.68	2.63

LGT: linagliptin; IMP 1: impurity 1; IMP 2: impurity 2; IMP 3: impurity 3.

**Table 2:** Percentage of degradation of LGT under different stress conditions to evaluate the selectivity of the analytical method.

Exposition time (h)	Conditions evaluated						
	UVA radiation	30% (v/v) hydrogen peroxide	Basic thermal (1 mol·L^−1^ at 80°C)	Acid thermal (1 mol·L^−1^ at 80°C)	Thermal (80°C)	Acid (1 mol·L^−1^)	Basic (1 mol·L^−1^)
0.25	—	—	17.14	—	—	—	—
0.5	—	—	31.26	—	—	—	—
0.75	—	—	44.24	—	—	—	—
1	4.36	—	58.74	—	9.37	—	—
1.5	—	—	74.14	—	—	—	—
2	7.75	6.87	86.57	9.04	10.00	—	—
3	13.56	—	—	—	8.31	—	—
4	34.86	8.65	—	9.89	—	—	—
6	—	8.73	—	11.11	—	—	—
10	—	9.85	—	15.50	—	—	—
24	—	—	—	—	—	3.12	12.06
48	—	—	—	—	—	—	23.19

**Table 3:** Intraday and interday precision of the analytical method.

	LGT		IMP 1		IMP 2		IMP 3	
	%	RSD	%	RSD	%	RSD	%	RSD
Intraday (*n*=6)								
Day 1	99.83	0.73	99.12	3.55	102.39	3.44	100.82	3.45
Day 2	99.57	1.33	99.78	3.00	101.76	4.52	101.31	4.63
Day 3	97.50	1.47	101.63	4.54	102.92	2.06	102.65	2.50
Interday (*n*=18)	98.97	1.36	100.17	4.14	102.35	3.50	101.59	4.08

LGT: linagliptin; IMP 1: impurity 1; IMP 2: impurity 2; IMP 3: impurity 3; RSD: relative standard deviation.

**Table 4:** Parameters evaluated and responses obtained on the robustness of LGT and synthetic impurities.

	Parameters				
	pH	Mobile phase	*T* (°C)	Flow rate	*λ*
LGT (%)	100.0	100.0	100.0	99.8	99.9
IMP 1 (%)	100.0	100.0	99.9	99.8	99.9
IMP 2 (%)	100.3	100.0	100.3	100.7	99.7
IMP 3 (%)	100.5	100.0	100.2	100.2	100.0

LGT: linagliptin; IMP 1: impurity 1; IMP 2: impurity 2; IMP 3: impurity 3; MP: mobile phase; *T* (°C): temperature; *λ*: wavelength.

**Table 5:** Results of potential risk to cause toxicity for linagliptin and impurities 1 to 3.

Toxicity risk	pKCSM	Osiris	LAZAR
Mutagenicity			
LGT	+	−	−
IMP 1	+	−	−
IMP 2	+	+ (Low)	+
IMP 3	−	−	−
Carcinogenicity			
LGT	NC^*∗*^	−	−
IMP 1	NC	−	−
IMP 2	NC	+ (High)	+
IMP 3	NC	−	−

^*∗*^NC: software does not calculate this property. Results are expressed as positive (+) or negative (−) and high, medium, and low for Osiris Property Explorer.

## Data Availability

The data used to support the findings of this study are included within the article in Section 3. The development of the analytical method describes all the tests performed to obtain the results found.
